# Relationship between lung function impairment, clinical characteristics and systemic inflammation based on a large-scale population screening

**DOI:** 10.3389/fmed.2025.1657151

**Published:** 2025-09-18

**Authors:** Xiaojun Ma, Yan Yu, Wenxia Guan, Shuming Guo, Zhancheng Gao, Mengtong Jin, Peng Liu, Lianyu Cheng, Chunting Chen, Kaiyu Ma, Yujie Zhou, Ran Li, Qi Wu

**Affiliations:** ^1^Department of Respiratory and Critical Care Medicine, Tianjin Medical University General Hospital, Tianjin Medical University, Tianjin, China; ^2^Department of Pulmonary and Critical Care Medicine, Linfen Central Hospital, Linfen, China; ^3^Linfen Clinical Medicine Research Center, LinFen Central Hospital, Linfen, China; ^4^Shanxi Provincial Clinical Medical Research Center for Respiratory Diseases (COPD), Linfen, China; ^5^Joint Research Center for Thoracic and Pulmonary Diseases, Shanxi Medical University, Linfen, China; ^6^Department of Respiratory and Critical Care Medicine, Peking University People’s Hospital, Beijing, China

**Keywords:** lung function impairment, preserved ratio impaired spirometry (PRISm), predictive model, population screening, early diagnosis, inflammatory biomarkers

## Abstract

**Background:**

Lung function impairment, a hallmark of chronic airway diseases like chronic obstructive pulmonary disease (COPD), is often underdiagnosed in China. Preserved Ratio Impaired Spirometry (PRISm) may represent an early, subclinical stage of this process. However, a comprehensive understanding of their clinical phenotypes, effective predictive strategies for early identification in large populations, and the role of systemic inflammation remains underexplored, particularly in the Chinese context. This study aimed to describe the clinical phenotypes of lung function impairment, identify predictive factors using machine learning, and explore associated systemic inflammation in a large-scale population screening.

**Methods:**

A prospective cross-sectional study was conducted in Hongtong County, China (2021–2024). Participants were classified into airflow obstruction, PRISm, and normal groups via portable spirometry. Using demographic, clinical, and laboratory data, we developed and validated several machine learning (ML) models to predict lung function impairment. Model performance was evaluated by the area under the receiver operating characteristic curve (AUC). Serum cytokines were measured by ELISA in matched sub-cohorts to assess systemic inflammation.

**Results:**

Among 9,284 enrolled adults, 51.0% had airflow obstruction, 6.7% had PRISm, and 42.3% were normal. We identified distinct phenotypes: the PRISm group was predominantly female with lower smoking rates but a higher risk of coronary heart disease. The airflow obstruction group was characterized by classical risk factors (older age, male sex, lower BMI, smoking) and specific renal and cerebrovascular comorbidities. The ML models identified older age, male sex, lower BMI, respiratory symptoms (cough, dyspnea), and higher creatinine and hemoglobin as key predictors, demonstrating modest performance with an AUC of 0.635 in the validation set. Immunologically, individuals with airflow obstruction or PRISm showed significantly lower serum IL-2 and higher IL-5 and IL-17A levels compared to controls.

**Conclusion:**

In a large-scale screening, individuals with airflow obstruction and PRISm present with distinct clinical phenotypes. A predictive model using simple clinical variables can help identify individuals at higher risk for lung function impairment, despite modest performance. Serum IL-2, IL-5, and IL-17A are potential biomarkers for the early recognition and understanding of airflow limitation.

## 1 Introduction

Lung function impairment represents a fundamental physiological aberration common to numerous chronic airway diseases, such as chronic obstructive pulmonary disease (COPD), asthma, bronchiectasis, and tuberculosis-ravaged lung. COPD remains a critical global health burden, ranking as the third leading cause of mortality worldwide (WHO, 2020) (1, 2). Updated 2023 data indicate COPD accounts for 6.4% of global deaths (1), with prevalence among Chinese adults ≥40 years reaching 13.7% (2012–2015) (3). Alarmingly, due to relatively low socioeconomic status and insufficient public awareness, especially in rural regions of China, less than 30% of cases are diagnosed early, and approximately 65% exhibit irreversible lung function decline at initial diagnosis (4). This high burden and low diagnosis rate highlight an urgent need for effective early identification and risk stratification strategies tailored for this vulnerable population.

In recent years, increased focus has been directed towards individuals who do not meet the spirometric criteria for persistent airflow obstruction yet display respiratory symptoms or diminished lung function. A noteworthy subgroup in this regard is Preserved Ratio Impaired Spirometry (PRISm), characterized by a preserved FEV1/FVC ratio (≥0.70) alongside a reduced FEV1 (<80% predicted) (5). PRISm is relatively common and has been linked to increased respiratory symptoms, exacerbations, cardiovascular comorbidities, and mortality compared to individuals with normal spirometry; however, its long-term progression and optimal management remain poorly understood (6–8). Several studies suggest that PRISm may signify an early stage of COPD or represent a distinct clinical phenotype (9–13).

Despite these advancements, a comprehensive understanding of the clinical heterogeneity of lung function impairment across its spectrum (from normal to PRISm and established obstruction), and the development of effective, practical predictive strategies for early identification in large populations, remains largely underexplored. Particularly, in high-prevalence, under-diagnosed rural populations like those in China, a systematic “comprehensive atlas” detailing distinct clinical phenotypes, associated predictive factors, and underlying biological mechanisms is still urgently needed. While conventional spirometry is the gold standard for diagnosis, its logistical impracticality and time-consuming nature present a significant barrier for large-scale population screening. To overcome this, the advent and validation of portable spirometers offer a crucial solution, enabling robust and feasible data collection in community settings (14).

Clinically, distinguishing between individuals with normal lung function, PRISm (subclinical), and advanced airflow obstruction is vital for implementing targeted prevention and management strategies. Although spirometry is fundamental, its results can be influenced by subjective factors. For instance, elderly individuals may have difficulty cooperating with lung function testing, while younger individuals are often unwilling to complete this time-consuming procedure. This variability underscores the need to find simpler, non-invasive factors correlated with lung function impairment. Integrating readily available blood test indicators with comorbidity metrics represents a crucial step toward developing effective predictive models. However, a more comprehensive understanding and robust risk stratification require moving beyond these isolated factors to incorporate multidimensional data, including demographic information, clinical history, laboratory markers, and environmental exposures (15–17). Analyzing such intricate and high-dimensional datasets presents a significant challenge, making machine learning techniques particularly well-suited for identifying complex patterns and constructing generalizable predictive models (18, 19).

Furthermore, the pathophysiology of lung function decline is deeply rooted in chronic inflammation (20). Neutrophilic inflammation was regarded as the key process in the pathogenesis of COPD. Systemic and airway-localized Th2 inflammation is characterized in a subgroup of COPD, usually with eosinophilia, served as a indicator for the usage of inhaled corticosteroid in the long-term management of COPD (21). While systemic inflammation is a known feature of COPD, its specific signature in the PRISm state, which may represent a transitional or distinct biological entity, remains poorly characterized (22). Investigating systemic inflammatory markers could therefore provide crucial insights into the processes that differentiate these early phenotypes from both normal lung function and established disease, thereby contributing to a more complete understanding of disease progression and guiding potential therapeutic targets.

Therefore, leveraging the feasibility of large-scale population screening using portable spirometry in a high-burden region of Shanxi Province, China, this study aimed to (1) comprehensively characterize the distinct clinical phenotypes of airflow obstruction and PRISm, (2) identify key clinical and laboratory predictive factors for lung function impairment using machine learning, and (3) explore associated systemic inflammatory profiles. By doing so, we seek to contribute to the early recognition and improved management strategies for chronic airway diseases in high-risk populations.

## 2 Methods and materials

### 2.1 Study design and population

This cross-sectional study was conducted between January 2021 and July 2024 as part of a large-scale epidemiological survey in the rural regions of Hongtong County, Linfen City, Shanxi Province, China. All adult residents aged 18 years and older were invited to participate. Participants were excluded if they met any of the following criteria: (1) inability to perform spirometry or failure to produce acceptable and repeatable results (quality grade below ‘A’ or ‘B’); (2) presence of an acute respiratory infection at the time of assessment; (3) current pregnancy; or (4) incomplete data, defined as >20% missing values for key variables.

Clinical data were systematically collected using an electronic questionnaire. This included demographic information [age, sex, Body Mass Index (BMI)], lifestyle factors (smoking status, biofuel and dust exposure), self-reported symptoms (chronic cough, sputum production, dyspnea, and chest pain), and the history of self-reported respiratory diseases and comorbidities (hypertension, chronic bronchitis, emphysema, COPD, asthma, lung cancer, other malignancies, tuberculosis, coronary heart disease, arrhythmia, diabetes mellitus, hyperthyroidism, and cerebrovascular disease). The study was approved by the ethics committee of Linfen Central Hospital (Ethics Approval No. 2021-1-1) and adhered to the principles of the Declaration of Helsinki. Informed consent was obtained from all participants prior to their involvement.

### 2.2 Spirometry and group classification

Given the nature of this large-scale population screening, all classifications were based on pre-bronchodilator spirometry. Pre-bronchodilator spirometry was performed on all participants using portable spirometry devices (BreathHome, Inc., China). To ensure quality control, all spirometry results were independently reviewed by two experienced pulmonologists. The following parameters were recorded: FEV1/FVC, FEV1, FVC, FEV1% predicted, FVC% predicted, forced expiratory flow at 25, 50, and 75% of FVC (FEF25, FEF50, FEF75), Forced Expiratory Time (FET), and FEF25-75. Quality grade of ‘A’ or ‘B’ was regarded to be eligible, which were defined as acceptable data in at least 2 repeated tests with repeatability difference less than 15%. Optimal results in repeated tests were utilized for further analysis. Based on these pre-bronchodilator results, participants were classified into three distinct groups:

- Airflow Obstruction Group: FEV1/FVC < 0.70
- PRISm Group: FEV1/FVC ≥ 0.70 and FEV1 < 80% predicted
- Normal Group: FEV1/FVC ≥ 0.70 and FEV1 ≥ 80% predicted

### 2.3 Laboratory test parameters

Routine assays of complete blood count, liver function, renal function, lipid profiles, and fasting glucose were performed for all the participants. Additionally, serum cytokine levels, including interleukin (IL)-2 (cat. no. 10353), IL-4 (cat. no. 10375), IL-5 (cat. no. 10376), IL-10 (cat. no. 13626), IL-17A (cat. no. 10344), and IL-22 (cat. no. 10356), were quantified by ELISA (LunChangShuoBiotech Inc., China) in matched cohorts. In consideration of IL-2 representing for Th1 inflammation, IL-4 and IL-5 for Th2 inflammation, IL-17A and IL-22 for Th17 pathway and neutrophil inflammation, and IL-10 for regulatory T cells, a comprehensive immunologic status can be evaluated. From the complete blood count data, several composite inflammatory indices were calculated: the neutrophil-to-lymphocyte ratio (NLR), neutrophil-to-eosinophil ratio (NER), systemic immune-inflammation index (SII = neutrophil × platelet/lymphocyte), systemic inflammation response index (SIRI = neutrophil × monocyte/lymphocyte), and the platelet-to-lymphocyte ratio (PLR).

### 2.4 Statistical analysis

All statistical analyses were performed using R software (version 4.4.1). Continuous variables with a normal distribution were presented as mean ± standard deviation (SD), while nonparametric variables were presented as median and interquartile range (IQR, 25th and 75th percentiles). Student’s *t*-test and analysis of variance with a *post hoc* Tukey HSD test were used for continuous parametric data, while continuous nonparametric data were analyzed using the Kruskal–Wallis test. Categorical variables were presented as counts (percentages) and were compared using the chi-square test or Fisher’s exact test, as appropriate.

In the training cohort, various multivariate modeling approaches were initially employed to identify predictive factors for airflow obstruction and PRISm. Specifically, LASSO regression with 10-fold cross-validation for optimal lambda selection, random forest (500 trees, max_depth = 15, min_samples_leaf = 5), and gradient boosting machine (1,000 boosting stages, learning_rate = 0.01, max_depth = 3) were used. Concurrently, adjusted odds ratios (ORs) with 95% confidence intervals (CIs) were provided by conventional logistic regression. Subsequently, using the selected features, four supervised learning algorithms were implemented to classify individuals with impaired versus normal lung function. These included logistic regression (L2 regularization, C = 1.0), random forest (1,000 trees, min_samples_split = 10), gradient boosting (2000 estimators, learning_rate = 0.005, with early stopping), and XGBoost (max_depth = 6, eta = 0.1, gamma = 0.5). Model performance was evaluated through stratified 5-fold cross-validation to ensure robustness.

A two-sided *p* value <0.05 was considered statistically significant.

## 3 Results

### 3.1 Comparison of clinical characteristics among individuals with airflow obstruction, PRISm and normal control

**Figure 1:**
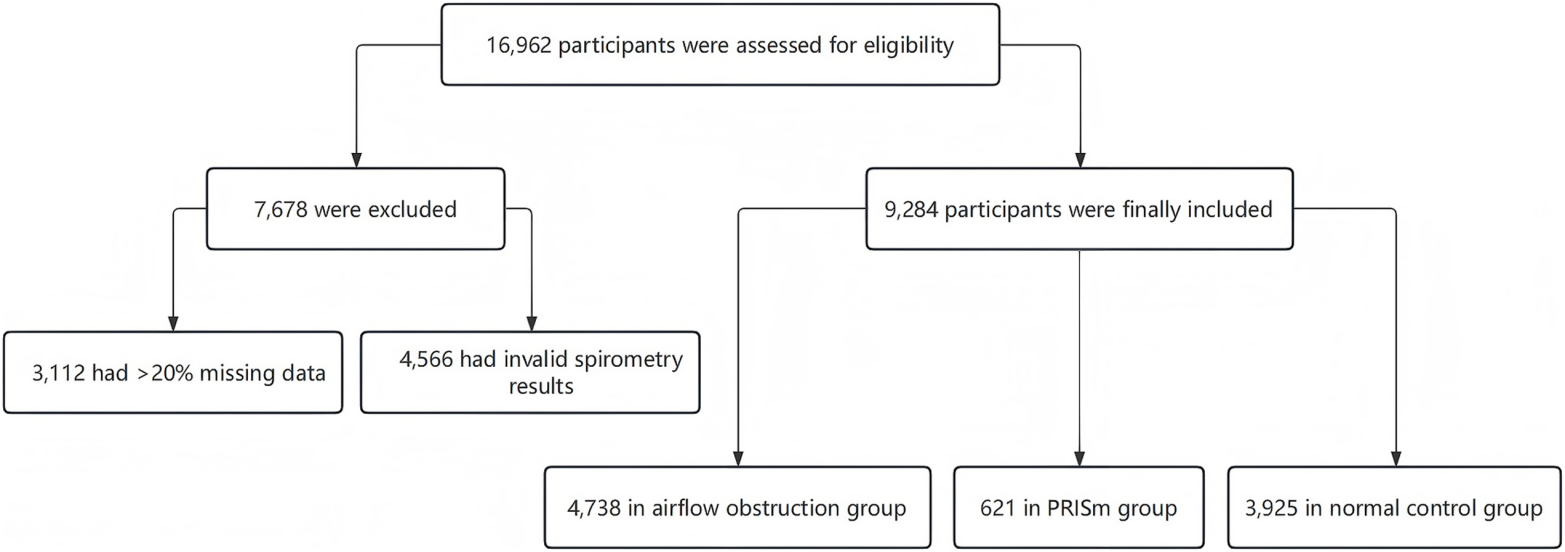
The flowchart of patient enrollment and follow-up classification.

A total of 16,962 participants were enrolled. After checking for missing data, a total of 9,284 participants were included in the final analysis, comprising 4,738 (51.0%) in the airflow obstruction group, 621 (6.7%) in the PRISm group, and 3,925 (42.3%) in the normal spirometry group (Figure 1).

Table 1 summarizes the important demographic and clinical characteristics of the study participants across the three groups. Participants with airflow obstruction were strikingly older [median (IQR): 62 (55–68) years] than both PRISm [55 (49–63) years] and normal groups [57 (50–63) years] (both *p* < 0.001). Furthermore, a significantly higher proportion of males characterized the airflow obstruction group compared to the other two groups. Participants in the airflow obstruction group exhibited a lower BMI than both the PRISm and normal groups.

**Table 1:** Baseline Characteristics among airflow obstruction, PRISm and normal groups.

Items	Airflow obstruction (*n* = 4,738)	PRSIm (*n* = 621)	Normal (*n* = 3,925)	*p* value
Age (IQR)	62 (55,68)	55 (49,63)	57 (50,63)	<0.001
Sex				<0.001
Male (%)	1988 (42.0%)	133 (21.4%)	1,154 (29.4%)	
Female (%)	2,750 (58.0%)	488 (78.6%)	2,771 (70.6%)	
BMI (IQR)	24.7 (22.6, 27.0)	25.5 (23.7, 27.8)	25.5 (23.5, 27.7)	<0.001
Smoking status				<0.001
Never (%)	2,886 (64.2%)	471 (83.1%)	2,729 (77.2%)	
Current (%)	1,262 (28.1%)	75 (13.2%)	642 (18.2%)	
Former (%)	348 (7.7%)	21 (3.7%)	164 (4.6%)	
Biofuel exposure (%)	2,768 (61.6%)	349 (61.6%)	2076 (58.7%)	0.031
Dust exposure (%)	524 (11.7%)	34 (6.1%)	322 (9.0%)	<0.001
Symptoms				
Chronic cough and sputum production (%)	1,097 (24.9%)	140 (24.4%)	814 (22.5%)	0.033
Dyspnea (%)	734 (16.3%)	68 (12.0%)	328 (9.3%)	<0.001
Chest pain (%)	169 (3.8%)	16 (2.8%)	133 (3.8%)	0.519
Self-reported disease				
Chronic bronchitis (%)	276 (6.1%)	21 (3.7%)	57 (1.6%)	<0.001
Emphysema (%)	69 (1.5%)	0 (0.0%)	6 (0.2%)	<0.001
COPD (%)	17 (0.4%)	0 (0.0%)	3 (0.1%)	0.013
Asthma (%)	186 (4.1%)	7 (1.2%)	22 (0.6%)	<0.001
Comorbidities				
Hypertension (%)	1,352 (30%)	180 (31.5%)	1,105 (31.1%)	0.497
Lung cancer (%)	10 (0.2%)	0 (0.0%)	0 (0.0%)	0.01
Former TB infection (%)	75 (1.7%)	2 (0.4%)	25 (0.7%)	<0.001
CHD (%)	188 (4.2%)	26 (4.6%)	92 (2.6%)	<0.001
Arrhythmia (%)	141 (3.1%)	16 (2.8%)	103 (2.9%)	0.813
Malignancy other than lung cancer (%)	14 (0.3%)	2 (0.4%)	5 (0.1%)	0.268
Diabetes (%)	284 (6.3%)	47 (8.3%)	229 (6.5%)	0.199
Cerebral infarction (%)	499 (11.1%)	47 (8.3%)	263 (7.4%)	<0.001

BMI: Body Mass Index; COPD: Chronic Obstructive Pulmonary Disease; CHD: Coronary Heart Disease.

Regarding lifestyle factors, the prevalence of current and former smoking was highest in the airflow obstruction group. Notably, the current smoking rate in airflow obstruction group (28.1%) was double that of the PRISm group (13.2%, *p* < 0.001). Dust exposure was also most prevalent in airflow obstruction group (11.7%), significantly high than in the PRISm (6.1%) and normal (9.0%) groups (*p* < 0.001).

Dyspnea was the predominant differentiating symptom, showing a clear gradient in prevalence: highest in the airflow obstruction group, followed by the PRISm group, and lowest in the normal group.

A significant discrepancy was observed between objective spirometry findings and self-reported diagnoses of respiratory diseases. Strikingly, only 0.4% of participants in the airflow obstruction group reported a prior diagnosis of COPD. The prevalence of self-reported asthma was highest in the airflow obstruction group compared to the other two groups.

Individuals with airflow obstruction exhibited a higher prevalence of self-reported prior tuberculosis and cerebrovascular diseases compared to the other groups. Coronary heart disease rates were also elevated in both the airflow obstruction (4.2%) and PRISm (4.6%) groups compared to the normal group (2.6%) (*p* < 0.001).

Higher counts of WBC and neutrophils were observed in both the airflow obstruction and PRISm group compared to the normal group. The highest levels of eosinophils and hemoglobin was shown in the airflow obstruction group. While the calculated inflammatory indices were less effective in discriminating between the impaired lung function groups, systemic inflammatory markers, including the NLR and SII, were significantly elevated in the airflow limitation group compared to the normal spirometry group (both *p* < 0.001). Furthermore, the airflow obstruction group exhibited higher levels of Blood Urea Nitrogen (BUN) and creatinine, and lower levels of Alanine Aminotransferase (ALT) and triglycerides than the other two groups. (Table 2).

**Table 2:** Laboratory test parameters among airflow obstruction, PRISm and normal groups.

Items	Airflow obstruction (*n* = 4,738)	PRSIm (*n* = 621)	Normal (*n* = 3,925)	*p* value
WBC × 10^9^/L (IQR)	5.99 (5.07, 7.11)	6.01 (4.96, 7.01)	5.81 (4.90, 6.84)	<0.001
Neutrophil × 10^9^/L (IQR)	3.33 (2.67, 4.14)	3.31 (2.66, 4.12)	3.18 (2.60, 3.96)	<0.001
Eosinophil × 10^9^/L (IQR)	0.11 (0.07, 0.18)	0.11 (0.07, 0.16)	0.10(0.07, 0.16)	<0.001
Valid counts (%)^ª^	4,650 (98.1)	607 (97.7)	3,850 (98.1)	
<0.1 × 10^9^/L (%)	1837 (39.5%)	262 (43.2%)	1738 (45.1%)	
0.1 ~ 0.3 × 10^9^/L (%)	2,410 (51.8%)	310 (51.1%)	1858 (48.3%)	
>0.3 × 10^9^/L (%)	403 (8.7%)	35 (5.8%)	254 (6.6%)	
Basophil × 10^9^/L (IQR)	0.03 (0.02, 0.04)	0.02 (0.02, 0.03)	0.02 (0.02, 0.03)	<0.001
Hemoglobin, g/L (IQR)	140 (130, 151)	136 (127, 144)	137 (128, 148)	<0.001
PLT × 10^9^/L (IQR)	236 (199, 278)	249 (207, 292)	239 (204, 281)	<0.001
SII (IQR)	390.85 (288.72, 530.83)	374.25 (287.74, 524.57)	379.94 (280.52, 512.09)	0.016
SIRI (IQR)	0.62 (0.43, 0.88)	0.62 (0.44, 0.89)	0.59 (0.42, 0.86)	0.040
PLR (IQR)	116.79 (92.52, 146.79)	120.10 (96.59, 151.89)	119.62 (95.97, 150.74)	<0.001
ALT, U/L (IQR)	18.90 (14.30, 26.20)	21.10 (15.40, 30.95)	20.10 (15.20, 28.50)	<0.001
Glucose, mmol/L (IQR)	4.79 (4.36, 5.36)	4.89 (4.51, 5.50)	4.81 (4.40, 5.35)	<0.001
BUN, mmol/L (IQR)	5.44 (4.65, 6.43)	5.06 (4.26, 6)	5.22 (4.43, 6.16)	<0.001
Creatinine, μmol/L (IQR)	59.40 (51.70, 69.00)	55.20 (48.00, 63.35)	57.40 (50.60, 66.50)	<0.001
Triglyceride, mmol/L (IQR)	1.42 (1.04, 1.98)	1.57 (1.18, 2.20)	1.50 (1.09, 2.12)	<0.001

ª Valid counts for eosinophil subgroups are shown. Percentages for these subgroups are calculated based on the number of participants with available eosinophil data, not the total group number.

Percentages may not sum to 100% due to rounding.

WBC, White Blood Cell; PLT, Platelets; SII, Systemic Immune-Inflammation Index; SIRI, Systemic Inflammation Response Index; PLR, Platelet-to-Lymphocyte Ratio; ALT, Alanine Aminotransferase; BUN, Blood Urea Nitrogen.

As expected from the group definitions, spirometric parameters showed a gradient of decline from the normal group to the PRISm and airflow obstruction groups. This trend was particularly evident in parameters of small airway function (FEF25, FEF50, and FEF75), which demonstrated a more pronounced stepwise deterioration across the groups. These small airway parameters also exhibited better sensitivity for identifying lung function impairment compared to conventional parameters such as FEV1/FVC, FEV1, and FVC (Table 3).

**Table 3:** Spirometry Parameters among airflow obstruction, PRISm and normal groups.

Items	Airflow obstruction (*n* = 4,738)	PRSIm (*n* = 621)	Normal (*n* = 3,925)	*p* value
FEV1/FVC (IQR)	61.92 (53.83, 66.46)	74.04 (71.87, 76.76)	75.21 (72.73, 78.18)	<0.001
FEV1, L/s (IQR)	2.00 (1.57, 2.43)	1.85 (1.61, 2.09)	2.46 (2.16, 2.88)	<0.001
FVC, L/s (IQR)	3.32 (2.73, 4.09)	2.46 (2.17, 2.78)	3.24 (2.84, 3.82)	<0.001
FEF25, L/s (IQR)	3.2 (2.16, 4.21)	3.91 (3.2, 4.71)	5.11 (4.33, 6.08)	<0.001
FEF50, L/s (IQR)	1.43 (0.95, 1.91)	2.05 (1.71, 2.41)	2.88 (2.38, 3.46)	<0.001
FEF75, L/s (IQR)	0.3 (0.21, 0.43)	0.45 (0.35, 0.58)	0.65 (0.48, 0.85)	<0.001
FEF25-75, L/s (IQR)	0.93 (0.61, 1.28)	1.46 (1.18, 1.76)	2.08 (1.7, 2.55)	<0.001

FEV1, Forced Expiratory Volume in 1 s; FVC, Forced Vital Capacity; FEF25, Forced Expiratory Flow at 25% of FVC; FEF50, Forced Expiratory Flow at 50% of FVC; FEF75, Forced Expiratory Flow at 75% of FVC; FEF25-75, Forced Expiratory Flow between 25 and 75% of FVC.

### 3.2 Predictive model for subclinical and advanced lung function impairment

To identify meaningful predictive factors for the early and precise recognition of lung function impairment, we combined the airflow obstruction group (representing advanced impairment) and the PRISm group (representing subclinical impairment) into a single “lung function impairment” entity (Group 1). This combined group was then compared against the normal control group (Group 2). The entire cohort was randomly divided into a training set and a validation set at a 70:30 ratio.

Univariate analysis of the training set was shown in Table 4. Group 1 exhibited a higher prevalence of tobacco and dust exposure, increased rates of chronic cough, sputum production, and dyspnea, and a greater proportion of individuals reporting a history of chronic respiratory diseases. Spirometry results revealed FEV1 and small airway functions parameters were distinguishable between Group 1 and Group 2, whereas FVC showed less discriminatory power.

**Table 4:** Univariate analysis of the training set.

Items	Group 1	Group 2	*p* value
	(*n* = 2,743)	(*n* = 2,493)	
Age (IQR)	56 (50, 61)	54 (48, 59)	<0.001
Sex			<0.001
Male (%)	985 (35.91%)	672 (26.96%)	
Female (%)	1758 (64.09%)	1821 (73.04%)	
BMI (IQR)	25.10 (23.05, 27.34)	25.61 (23.71, 27.77)	<0.001
Smoking status			<0.001
Never (%)	1907 (69.52%)	1943 (77.94%)	
Current (%)	683 (24.90%)	459 (18.41%)	
Former (%)	153 (5.58%)	91 (3.65%)	
Biofuel exposure (%)	1611 (58.73%)	1417 (56.84%)	0.166
Dust exposure (%)	332 (12.10%)	234 (9.39%)	0.002
Symptoms			
Chronic cough and sputum production (%)	712 (25.96%)	555 (22.26%)	0.002
Dyspnea (%)	400 (14.58%)	236 (9.47%)	<0.001
Chest pain (%)	101 (3.68%)	93 (3.73%)	0.926
Self-reported disease			
Chronic bronchitis (%)	138 (5.03%)	30 (1.20%)	<0.001
Comorbidities			
Cerebral infarction (%)	219 (8%)	125 (5%)	<0.001
Laboratory examination			
WBC × 10^9^/L (IQR)	5.98 (5.04, 7.09)	5.84 (4.91, 6.88)	<0.001
Monocyte×10^9^/L (IQR)	0.39 (0.31, 0.49)	0.36 (0.28, 0.45)	<0.001
Eosinophil×10^9^/L (IQR)	0.11 (0.07,0.18)	0.1 (0.06, 0.16)	<0.001
Hemoglobin, g/L (IQR)	139 (129.5, 151)	138 (128, 149)	<0.001
NER (IQR)	30.44 (18.58, 49.17)	28.82 (18.23, 47.29)	0.031
BUN, mmol/L (IQR)	5.25 (4.46, 6.15)	5.1 (4.33, 6)	<0.001
Creatinine, μmol/L (IQR)	57 (50, 66.3)	56 (49.4, 65)	0.034
Triglyceride, mmol/L (IQR)	1.45 (1.04, 2.06)	1.51 (1.09, 2.16)	0.002
Spirometry examination			
FEV1/FVC % (IQR)	64.38 (56.97, 68.52)	75.61 (73.15, 78.5)	<0.001
FEV1, L/s (IQR)	2.11 (1.74, 2.55)	2.52 (2.22, 2.95)	<0.001
FEF25, L/s (IQR)	3.61 (2.63, 4.56)	5.21 (4.45, 6.16)	<0.001
FEF50, L/s (IQR)	1.68 (1.20, 2.18)	2.98 (2.5, 3.55)	<0.001
FEF75, L/s (IQR)	0.35 (0.25, 0.5)	0.68 (0.51, 0.88)	<0.001
FEF25-75, L/s (IQR)	1.13 (0.76, 1.48)	2.18 (1.8, 2.63)	<0.001

BMI, Body Mass Index; WBC, White Blood Cell; NER, Neutrophil-to-eosinophil ratio; BUN, Blood Urea Nitrogen; FEV1, Forced Expiratory Volume in 1 s; FVC, Forced Vital Capacity; FEF25, Forced Expiratory Flow at 25% of FVC; FEF50, Forced Expiratory Flow at 50% of FVC; FEF75, Forced Expiratory Flow at 75% of FVC; FEF25-75, Forced Expiratory Flow between 25 and 75% of FVC.

To identify robust predictive factors for lung function impairment, several machine learning algorithms for modeling were employed, including logistic regression, Random Forest (RF), Gradient Boosting Machine (GBM) and XGBoost. The validation set was served as an independent external validation for model performance.

**Figure 2:**
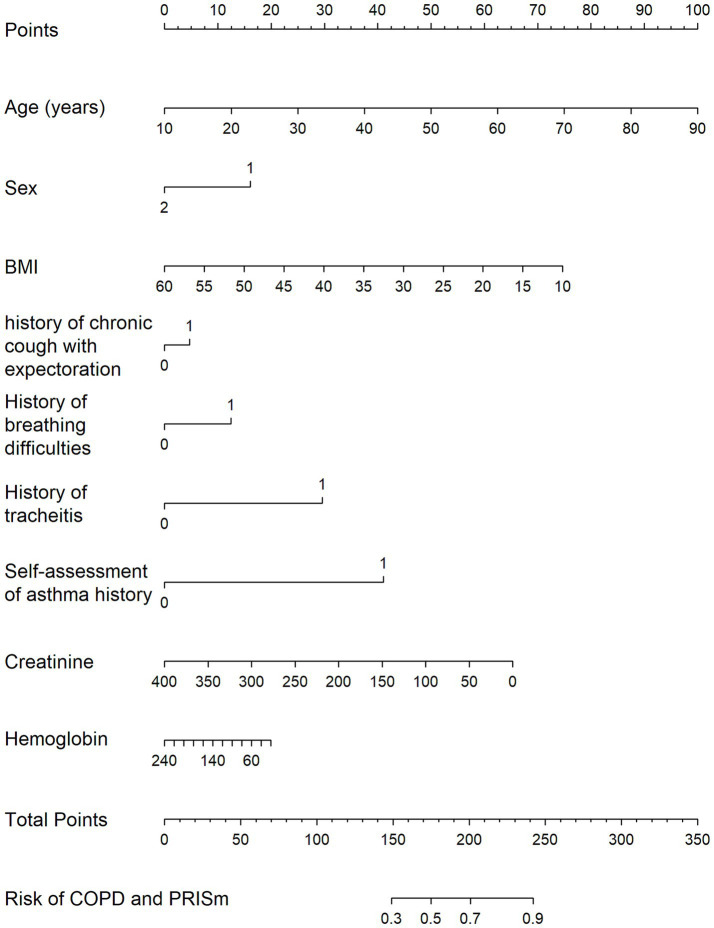
Predictive factors of lung function impairment by multivariable logistic regression analysis.

**Figure 3:**
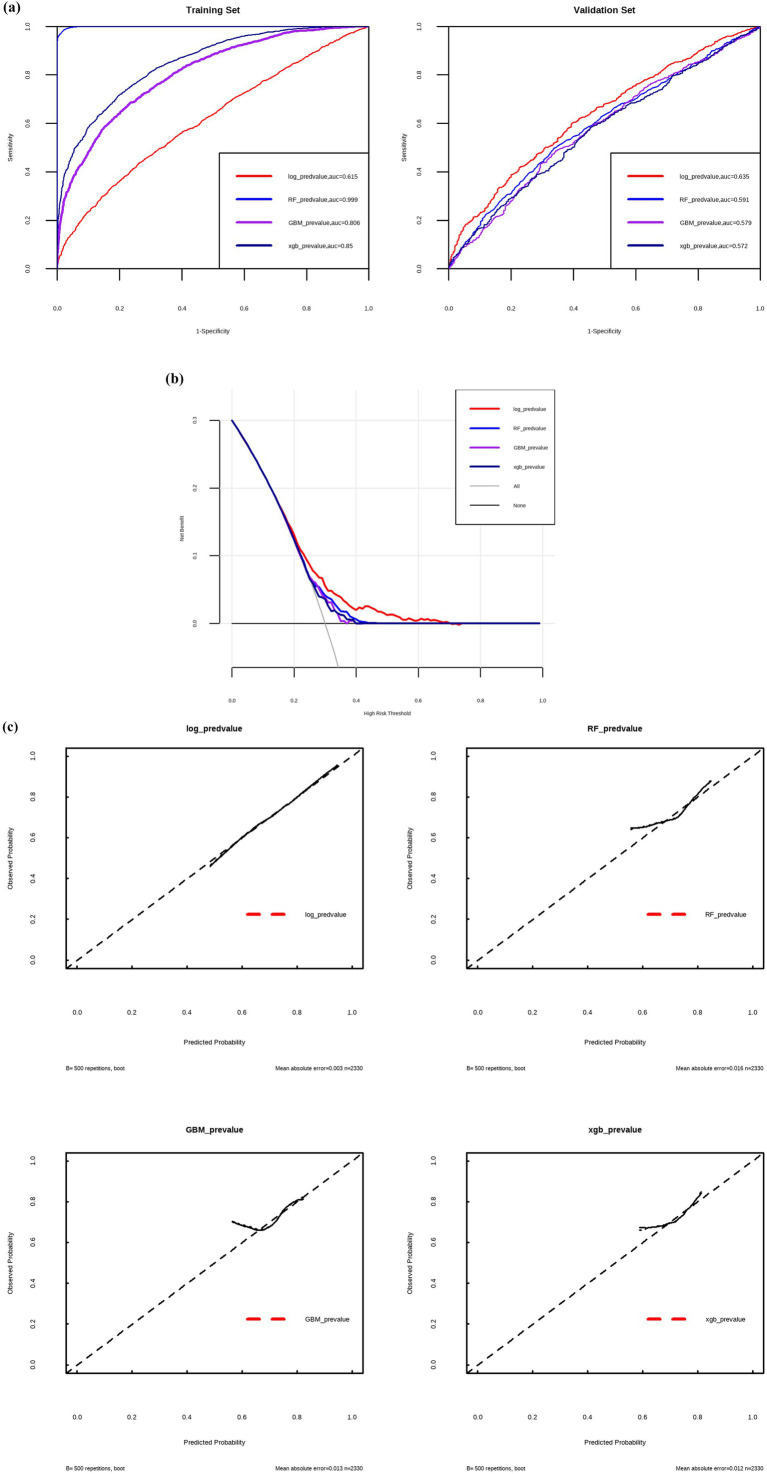
ROC-AUC curves **(a)**, clinical decision curves **(b)** and calibration curves **(c)** to evaluate the predictive efficiency by different models. Log, logistic regression; RF, random forest; GBM, gradient boosting machine; xgb, XGBoost.

Multivariable logistic regression analysis identified multiple predictive factors, including older age, male sex, lower BMI, chronic cough and sputum, dyspnea, a history of chronic bronchitis and asthma, higher creatinine, and higher hemoglobin level (Table 5; Figure 2). The logistic model was evaluated to be the optimal model rather than RF, GBM and xgb models, with an area under the curve (AUC) of 0.615 in the training set and 0.635 in the validation set (Figure 3).

**Table 5:** Predictive factors for lung function impairment using logistic regression modeling.

Independent var	B	SE	*z*	*p* value	OR [95%CI]
Constant	1.312	0.466	2.816	0.005	
Age	0.027	0.003	7.817	0.000	1.03 [1.02, 1.03]
Female	−0.514	0.085	−6.028	0.000	0.6 [0.51, 0.71]
BMI	−0.045	0.008	−5.353	0.000	0.96 [0.94, 0.97]
Chronic cough and sputum	0.224	0.065	3.429	0.001	1.25 [1.1, 1.42]
Dyspnea	0.285	0.091	3.138	0.002	1.33 [1.11, 1.59]
Self-reported chronic bronchitis	0.998	0.206	4.850	0.000	2.71 [1.81, 4.06]
Self-reported asthma	1.556	0.307	5.062	0.000	4.74 [2.59, 8.65]
Creatinine	−0.006	0.002	−2.435	0.015	0.99 [0.99, 1]
Hemoglobin	−0.003	0.002	−1.522	0.128	1 [0.99, 1]

BMI, Body Mass Index.

### 3.3 Comparison of systemic inflammation among lung function groups

After matching for age, sex and smoking status, we quantified six key cytokines in a sub-cohort consisting of 202 participants with airflow obstruction, 202 participants in PRISm, and 209 participants in normal group (Table 6).

**Table 6:** Serum cytokine Levels among airflow obstruction, PRISm and normal groups in matched sub-cohorts.

Cytokines	Airflow obstruction	PRISm	Normal control	*p* value
	(*n* = 202)	(*n* = 202)	(*n* = 209)	
IL-2 (IQR)	162.52 (106.92, 239)	240.13 (179.35, 296.67)	280.17 (183.36, 372.2)	<0.001
IL-4 (IQR)	26.39 (15.88, 33.82)	17.73 (15.86, 25.85)	25.84 (17.67, 33.18)	<0.001
IL-5 (IQR)	94.29 (59.5, 163.56)	93.82 (80.59, 108.35)	62.69 (56.89, 69.21)	<0.001
IL-10 (IQR)	39.35 (30.01, 47.69)	28.68 (20.92, 37.31)	44.26 (27.8, 54.83)	<0.001
IL-17A (IQR)	9.49 (6.77, 13.05)	9.42 (7.05, 13.22)	7.95 (5.81, 10.98)	0.001
IL-22 (IQR)	199.56 (126.38, 265.49)	202.56 (131.1, 286.45)	183.37 (133.37, 227.14)	0.03

IL-2, Interleukin-2; IL-4, Interleukin-4; IL-5, Interleukin-5; IL-10, Interleukin-10; IL-17A, Interleukin-17A; IL-22, Interleukin-22.

While statistically significant differences were observed for all measured cytokines across the three groups, some did not demonstrate a clear or clinically relevant gradient. Upon pairwise comparison, IL-2 levels showed a significant gradient, with the lowest levels in the airflow obstruction group, intermediate levels in the PRISm group, and the highest levels in the normal group (airflow obstruction < PRISm < Normal). Notably, the levels of IL-5 and IL-17A were significantly elevated in the combined lung function impairment group (Group 1: airflow obstruction and PRISm) compared to the normal control group (Group 2). These findings suggest that IL-2, IL-5 and IL-17A could serve as potential biomarkers for the identification of lung function impairment.

## 4 Discussion

This large-scale, real-world population screening characterized the distinct clinical, metabolic, and inflammatory phenotypes of airflow obstruction and PRISm in rural China, a region with a significant COPD burden (3). We identified a high prevalence of undiagnosed lung function impairment (51.0% airflow obstruction; 6.7% PRISm), reflecting a profound discrepancy between objective spirometric abnormalities and self-reported disease. Only 0.4% of participants with airflow obstruction reported a prior COPD diagnosis, highlighting the critical need for population-based screening initiatives. The diagnosis of chronic bronchitis, COPD, asthma and bronchiectasis was widely confounded in the underdeveloped regions of China. Therefore, simplified predictive model in our study which could be easily obtained from questionnaire and regular blood tests can prompt the identification of population with airflow limitation. Recommendations for these high-risk or undiagnosed individuals can be made for further spirometry, CT scan and other clinical examinations to define the accurate diagnosis of chronic airway diseases.

Our study provides an in-depth characterization of PRISm as a distinct clinical entity. Unlike the airflow obstruction group, PRISm was more prevalent in females, associated with lower smoking and dust exposure rates, and exhibited symptom burden intermediate to normal and obstructed groups. Notably, PRISm showed a unique comorbidity pattern, including an elevated risk of coronary heart disease and a characteristic atherogenic lipid profile (higher triglycerides), suggesting intrinsic metabolic dysregulation as a potential underlying mechanism that warrants further investigation. In contrast, the airflow obstruction group presented with a classical risk profile tied to smoking and dust exposure, a higher burden of respiratory symptoms, and comorbidities like tuberculosis and cerebrovascular disease.

We identified several readily available clinical and laboratory markers with predictive value for lung function impairment, consistent with some previous literature (11, 23–25). The predictive associations with metabolic (e.g., lower ALT, higher creatinine) and hematological indices (e.g., higher hemoglobin) reinforce the systemic nature of these conditions, suggesting that metabolic dysfunction and chronic hypoxia may be key pathophysiological drivers. Immunologically, individuals with lung function impairment (airflow obstruction or PRISm) exhibited elevated serum IL-5 and IL-17A, alongside lower IL-2 levels. This cytokine profile, coupled with cellular findings (elevated neutrophils and eosinophils in airflow obstruction), suggests the predominance of Th2 and Th17-driven inflammation over Th1 pathways in the pathogenesis of airflow limitation. These findings contribute to the complex and heterogeneous landscape of inflammatory pathways in chronic airway diseases (22, 26–29), highlighting specific targets for future mechanistic studies.

Our study has several limitations. Its cross-sectional design allows for identification of associations and predictive factors, but not causality; longitudinal follow-up is required to confirm true predictors of disease progression. Second, our reliance on pre-bronchodilator spirometry, while pragmatic for large-scale screening and supported by its utility for risk stratification (30), limits our ability to definitively differentiate between reversible (e.g., asthma) and persistent (e.g., COPD) airflow limitation. Third, cytokine measurements were performed on a smaller, matched sub-cohort, which, while controlled for confounders, limited statistical power for inclusion in the overall predictive model.

Future research should focus on validating these distinct phenotypes and the predictive model in diverse independent cohorts, particularly through prospective longitudinal studies. Further investigation is also warranted to elucidate the precise mechanistic roles of serum IL-2, IL-5, and IL-17A in the early pathogenesis of lung function impairment and their potential as prognostic biomarkers or therapeutic targets. Finally, exploring the integration of additional, readily available clinical or biological parameters, and potentially more advanced machine learning approaches, could further enhance the predictive power of such models.

## 5 Conclusion

In summary, this large-scale, real-world screening study reveals the distinct clinical, metabolic, and inflammatory landscapes of airflow obstruction and PRISm, highlighting a substantial burden of undiagnosed lung function impairment. We established a potential predictive model using simple clinical history and routine laboratory assays that could help identify high-risk individuals for targeted, early spirometric screening. Furthermore, our findings suggest that serum IL-2, IL-5 and IL-17A may serve as biomarkers for airflow limitation, providing a deeper understanding of the immunological mechanisms driving the gradual impairment of lung function. These insights can inform public health strategies aimed at the early detection and management of chronic airway diseases.

## Data Availability

The datasets presented in this study can be found in online repositories. The names of the repository/repositories and accession number(s) can be found in the article/supplementary material.

## Glossary

ALT: Alanine Aminotransferase
BMI: Body Mass Index
BUN: Blood Urea Nitrogen
CHD: Coronary heart disease
COPD: Chronic Obstructive Pulmonary Disease
FEF25: Forced Expiratory Flow at 25% of FVC
FEF50: Forced Expiratory Flow at 50% of FVC
FEF75: Forced Expiratory Flow at 75% of FVC
FEF25–75: Forced Expiratory Flow between 25 and 75% of FVC
FET: Forced expiratory time
FEV1: Forced expiratory volume in the first 1.0 s
FVC: Forced vital capacity
IL-2: Interleukin-2
IL-4: Interleukin-4
IL-5: Interleukin-5
IL-10: Interleukin-10
IL-17A: Interleukin-17A
IL-22: Interleukin-22
ML: Machine Learning
NER: Neutrophil-to-eosinophil ratio
NLR: Neutrophil-to-lymphocyte ratio
PLR: Platelet-to-lymphocyte ratio
PLT: Platelets
PRISm: Preserved Ratio Impaired Spirometry
SIRI: Systemic inflammation response index
SII: Systemic immune-inflammation index
TB: Tuberculosis
WBC: White blood cells
